# Umbilical Cord Wharton’s Jelly Repeated Culture System: A New Device and Method for Obtaining Abundant Mesenchymal Stem Cells for Bone Tissue Engineering

**DOI:** 10.1371/journal.pone.0110764

**Published:** 2014-10-20

**Authors:** Zhengqi Chang, Tianyong Hou, Junchao Xing, Xuehui Wu, Huiyong Jin, Zhiqiang Li, Moyuan Deng, Zhao Xie, Jianzhong Xu

**Affiliations:** 1 National & Regional United Engineering Lab of Tissue Engineering, Department of Orthopaedics, Southwest Hospital, the Third Military Medical University, Chongqing, China; 2 Center of Tissue Engineering Research and Application, the Third Military Medical University, Chongqing, China; 3 Laboratory of Tissue Engineering, Chongqing City, Chongqing, China; 4 Department of Orthopedics, General Hospital of Jinan Military Commanding Region, Jinan, China; Georgia Regents University, United States of America

## Abstract

To date, various types of cells for seeding regenerative scaffolds have been used for bone tissue engineering. Among seed cells, the mesenchymal stem cells derived from human umbilical cord Wharton’s jelly (hUCMSCs) represent a promising candidate and hold potential for bone tissue engineering due to the the lack of ethical controversies, accessibility, sourced by non-invasive procedures for donors, a reduced risk of contamination, osteogenic differentiation capacities, and higher immunomodulatory capacity. However, the current culture methods are somewhat complicated and inefficient and often fail to make the best use of the umbilical cord (UC) tissues. Moreover, these culture processes cannot be performed on a large scale and under strict quality control. As a result, only a small quantity of cells can be harvested using the current culture methods. To solve these problems, we designed and evaluated an UC Wharton’s jelly repeated culture device. Using this device, hUCMSCs were obtained from the repeated cultures and their quantities and biological characteristics were compared. We found that using our culture device, which retained all tissue blocks on the bottom of the dish, the total number of obtained cells increased 15–20 times, and the time required for the primary passage was reduced. Moreover, cells harvested from the repeated cultures exhibited no significant difference in their immunophenotype, potential for multilineage differentiation, or proliferative, osteoinductive capacities, and final osteogenesis. The application of the repeated culture frame (RCF) not only made full use of the Wharton’s jelly but also simplified and specified the culture process, and thus, the culture efficiency was significantly improved. In summary, abundant hUCMSCs of dependable quality can be acquired using the RCF.

## Introduction

Tissue-engineered bones (TEBs) have been widely researched for facilitating bone repair, within a broad range of clinical applications [1], [2], [3]. Due to their self-renewal and multipotent capacities, mesenchymal stem cells (MSCs) are considered the ‘gold standard’ seed cells for constructing TEBs. To date, MSCs derived from bone marrow and adipose tissue have been widely applied in bone tissue engineering [4], [5]. However, there are still some drawbacks regarding the applications of bone marrow- or adipose-derived mesenchymal stem cells (BMSCs or ADSCs), including the low number of MSCs in marrow or fat, and the fact that with aging, their proliferative and differentiation capacities decrease significantly [6]. Moreover, the procurement of MSCs involves an invasive and uncomfortable extraction procedure, leading to potential complications and morbidity [7]. Generally, successful tissue engineering applications require large numbers of MSCs, particularly in orthopedics (in the present study, cells were seeded in DBM at a density of 4×10^6^/cm^3^). Thus, seeking alternative types of MSCs, with wider availability, an easier harvesting procedure, and more robust proliferative and differentiation abilities has become an urgent issue.

MSCs derived from human umbilical cord Wharton’s jelly (hUCMSCs) represent a prospective cell source and hold tremendous promise for tissue engineering [8], [9], [10]. Compared with BMSCs and ADCS, they exhibit considerable advantages, such as the lack of ethical controversies, accessibility, extraction procedures that are painless for donors, a reduced risk of contamination, osteogenic differentiation capacities, and higher immunomodulatory capacity. [11], [12]. In addition, there are relatively few ethical restrictions or medico-legal limitations on extracting and applying these cells [13]. More importantly, the ample resources of the cords and the feasible cryopreservation of hUCMSCs allow these cells to be banked for future tissue engineering applications [6]. Therefore, techniques aimed at maximizing the number of hUCMSCs isolated from umbilical cords (UCs) are extremely valuable.

Various approaches have been designed and evaluated for hUCMSC isolation. Currently, enzymatic digestion with trypsin and/or collagenase is associated with rapid isolation but a limited number of cells; even more unfortunate is that the enzymes may impair cell junction and may alter the cells’ immunophenotype [14]. Moreover, the enzymatic potency, digestion period, and temperature complicate the quality control of the dissociation procedure. In contrast, the method of explant culture entails relatively uniform manipulation, has a higher success rate, and can provide more cells [15]. However, this approach calls for longer cultivation periods, and most of the hUCMSCs residing in the umbilical cord cannot be extracted due to the loose attachment of the explant.

In this study, we designed and evaluated an umbilical cord Wharton’s jelly repeated culture system aimed at increasing the utilization of umbilical cords and the amount of accessible hUCMSCs during isolation and culture. To verify the cyto-homogeneity of the repeated cultures, the characteristics of the hUCMSCs obtained from the first, third and fifth isolation and culture were determined, including their phenotypes and their proliferative, multipotent differentiation abilities. In addition, their osteogenic capacities *in vitro* and *in vivo* were assessed and compared.

## Materials and Methods

### Ethics statement

This study was carried out in strict accordance with the recommendations in the Guide for the Care and Use of Laboratory Animals of the National Institutes of Health. The protocol was approved by the Laboratory Animal Welfare and Ethics Committee of the Third Military Medical University in Chongqing, China (Permit Number: SYXK-PLA-2007035). All surgery was performed under sodium pentobarbital anesthesia, and all efforts were made to minimize suffering. The human umbilical cords were harvested from the discarded tissue of puerperants undergoing surgery under the conditions of the patients’ written informed consent and approval of Southwest Hospital Ethics Committee.

### Distribution of MSCs in the human umbilical cord

To reveal the distributive changes of MSCs in the human umbilical cord, immunofluorescence (IF) and hematoxylin and eosin (HE) staining were performed. The samples included UCs obtained from patients undergoing surgery (with the patients’ informed consent, approved by Southwest Hospital Ethics Committee). A part of the samples was removed and fixed in a solution of 4% paraformaldehyde at room temperature (RT) for 20****minutes. Then, 6-µm transverse cryosections were cut using a cryostat (LEICA CM3050S, Leica, Wetzlar, Germany) for IF and HE staining. After blocking with normal donkey serum (1∶20) for 30****min at RT, the sections were incubated with the following primary antibodies overnight at 4°C: anti-CD73 (1∶50, goat, Santa Cruz Biotechnology, Santa Cruz, CA, USA), anti-CD90 (1∶50, goat, Santa Cruz Biotechnology), anti-CD105 (1∶50, goat, Santa Cruz Biotechnology, Santa Cruz, CA, USA), or anti-CD31 (1∶20, rabbit, Abcam, Cambridge, UK). The secondary antibodies used were donkey anti-goat-TRITC (1∶100) and donkey anti-rabbit-Cy3 (1∶50; both from Jackson ImmunoResearch, USA). The sections were counterstained with DAPI, and the images were captured using a confocal laser scanning microscope (CLSM; Leica Biosystems, Wetzlar, Germany). Other portions of the UC samples were cut into small fragments (1×1×2****mm^3^), harvested after 7 days of suspension culturing, and then cultured adherently for 7 days in the novel culture dish described below. Subsequent IF staining was performed as described above.

### Isolation and culture of hUCMSCs

**Figure 1:**
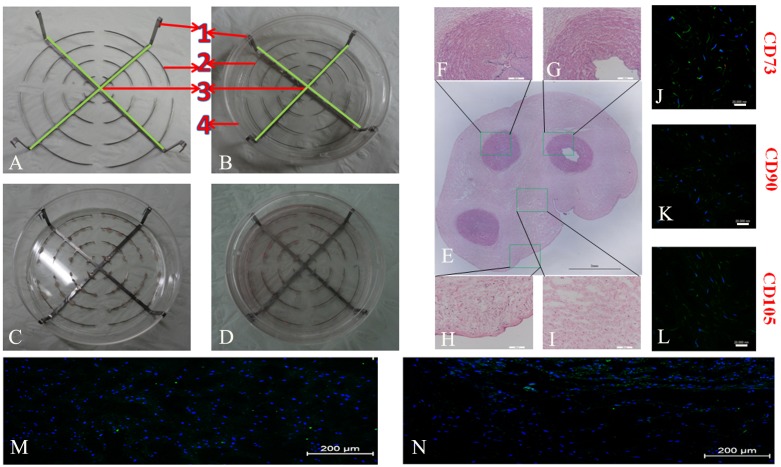
The repeated culture frame and structure of umbilical cord. The repeated culture frame is shown in figures A and B, and figures C and D display in cultures. HE stained cross-section of an umbilical cord (E) shows the veins (F), arteries (G), amnion layer (H) and Wharton’s jelly (I), respectively. The right panel shows the IF staining of CD73 (J), CD90 (K), and CD105 (L) in the Wharton’s jelly. Figures M and N show uniform cell distribution in the suspended tissue block and cell distribution owards the bottom in adherent culture for 7 days, respectively. Scale bars: 2****mm in E; 200 um in F–I; 20 um in J–L; and 200 um in M and N.

A novel culture system was made by modifying conventional culture dishes (CCDs) and was applied to repeated explant culture. As shown in Fig. 1A, the repeated culture frame (RCF) was composed of 4 parts: (1) four hooks for clamping, (2) concentric steel rings with intervals between each piece (0.2-mm wide), (3) a bracket body fabricated from two mutually perpendicular steel shims (1-mm wide), and (4) a dish (10****cm in diameter) with four slots. Human UCMSCs were isolated from the UCs of three normal full-term newborns (39–40 weeks) from patients who underwent cesarean section and had signed consent forms, according to the methods described previously [16]. The tissues (5****cm) proximal to the placenta were excised from each UC, and the internal vessels were extensively washed with PBS solution and then removed using a toothed microforceps from one end to the other under the help of Operation Magnifier (2.5x, Changzhou Jinyang Medical Instrument Ltd.). The remaining tissues were diced into small fragments (1×1×2****mm^3^), which were washed by PBS solution and centrifuged times at a force of 450****g in 4°C for 10****minutes, and the process was repeated 3 times. Then, 50 tissue blocks were strung on the steel rings and put in 90****mm culture dish (Corning, USA), followed by a 4-hour incubation in a humidified 37°C/5% CO_2_ incubator and addition of a small amount of nutrient medium, containing Dulbecco’s modified Eagle’s medium/Hamm’s Nutrient Mixture F12 (1∶1) (DMEM/F12, Hyclone) with 10% foetal bovine serum (FBS, Hyclone), 100****U/mL penicillin (Sigma, St. Louis, MO) and 100****µg/mL streptomycin (Sigma, St. Louis, MO). The residual tissue block were transferred into a same culture dish at the same density and manipulation as follows. They were left undisturbed for 2 hours in a humidified 37°C/5% CO_2_ incubator, moistened by few common medium, and inverted for another 2-hour period by reversing culture dish for increasing tissue adherence. Finally, eight ml common media was added in all culture dishes for both culture methods. Then, the explants were routinely cultured and the common medium was replaced every 2 days. The number of failed (floating) and successful (adherent) tissue block, around which cells were observed on the bottom of the dish, in both dishes were recorded *omnibus bidendis* until the 12th day. After 12 days of culture, the numbers of cells in both types of dishes were counted. Then, the cells were passaged and the bracket, together with the concatenated tissue block, was transferred into another modified culture dish (with slots) for repeated culture (Fig. 1A). As such, the culture was repeated 5 times.

### Cell characterization

Passage 3 cells from the 1^st^, 3^rd^, and 5^th^ cultures, designated as T1-P3, T3-P3, and T5-P3 cells, respectively, were characterized by flow cytometric immunophenotyping (FCI) and assessment of their multilineage differentiation potential. Briefly, approximately 1×10^6^ cells in separate 100-µl aliquots were labeled with the fluorochrome-conjugated antibodies. The antibodies used were specific for CD44, CD73, CD90, CD105, CD34 and CD45; the recommended isotype control for each fluorochrome (BD Biosciences, USA) was also used. The cells were analyzed using BD FACS Diva software.

To assess their multiple differentiation potential, T1-P3, T3-P3, and T5-P3 cells were seeded in three 6-well plates at a density of 5,000/cm^2^ and osteogenic, adipogenic, or chondrogenic media (all purchased from Hyclone) were added. The media were replaced every 2 days. After 21 days, the osteogenic, adipogenic, and chondrogenic differentiation potential was verified by staining with alizarin red, Oil Red O, and Alcian blue, respectively. In parallel, cells were harvested at designated time points (0, 7, 14 and 21 days) and the total RNA was extracted using a Qiagen RNeasy Mini Kit (Qiagen, USA). The quality and concentration of the RNA were quantified by spectrophotometry. The cDNA was prepared from 1****µg of total RNA using a cDNA synthesis kit according to the manufacturer’s instructions (Promega, Madison, WI, USA). Real-time PCR was performed using the SYBR ExScript RT-PCR kit (Perfect Real Time; TaKaRa, Dalian, China). GAPDH was used as the internal control. The amplification conditions were as follows: 94°C for 3****min, followed by 40 cycles of 95°C for 15****s, 94°C for 30****s, and 72°C for 1****min. The RT-PCR products (5****µl) were electrophoresed on a 2% agarose gel, and the bands were visualized by ethidium bromide staining. The differentiation markers and their corresponding primer sets are shown in Table 1-part 1.

**Table 1:** Primers Used for RT-PCR and Real-Time RT-PCR.

Part 1. Primers Used for RT-PCR				
Gene	Forward primer (5′-3′)	Reverse primer (5′-3′)	bp	Acc. No
**ALP**	ACGTGGCTAAGAATGTCATC	CTGGTAGGCGATGTCCTTA	475	NM_000478
**RUNX2**	CCACCCGGCCGAACTGGTCC	CCTCGTCCGCTCCGGCCCACA	238	NM_004348
**ON**	ATGAGGGCCTGGATCTTCTT	GCTTCTGCTTCTGAGTCAGA	580	NM_003118
**COL II**	TTTCCCAGGTCAAGATGGTC	TCACCTGGTTTTCCACCTTC	498	NM_033150
**COL X**	GCCCAAGAGGTGCCCTGGAATAC	CCTGAGAAAGAGGAGTGGACATAC	703	NM_000493
**ACAN**	TGAGGAGGGCTGGAACAAGTACC	GGAGGTGGTAATTGCAGGGAACA	395	NM_013227
**LPL**	GTCCGTGGCTACCTGTCAT	AGCCCTTTCTCAAAGGCTTC	717	NM_000237
**Adipsin**	GGTCACCCAAGCAACAAAGT	CCTCCTGCGTTCAAGTCATC	272	NM_001982
**PPAR-γ**	GGAAAGACAACAAACAAATCAC	TGCATTGAACTTCACAGCAAAC	414	NM_005037
β**-actin**	CTTAGTTGCGTTACACCCTTTCTTG	CTGCTGTCACCTTCACCGTTCC	159	NM_001101
**Part 2. Primers Used for Real-Time RT-PCR**				
**ALP**	CCCACAATGTGGACTACCT	GAAGCCTTTGGGGTTCTTC	143	NM_000478
**RUNX2**	CGGAGTGGACGAGGCAAGAG	TGAGGAATGCGCCCTAAATC	194	NM_004348
OC	CACTCCTCGCCCTATTGGC	CCCTCCTGCTTGGACACAAAG	112	NM_199173
**18S**	GTAACCCGTTGAACCCCATT	CCATCCAATCGGTAGTAGCG	151	NR_003286

ALP, alkaline phosphatase; RUNX2, runt-related transcription factor 2; ON, osteonectin; COL II, type II collagen; COL X, type X collagen; ACAN, aggrecan; LPL, lipoprotein lipase; PPAR-γ, peroxisome proliferator-activated receptor γ; OC, osteocalcin.

### Evaluation of the population doubling time

The growth characteristics of the cells from the repeated culture were evaluated. T1-P3, T3-P3, and T5-P3 cells were seeded in 24-well plates with common medium at a density of 1×10^4^ cells per plate at day 0, and cells were counted every day until day 10. The average number of cells was calculated from three replicates. The growth curves of the cell cultures were plotted, and the population doubling time (PDT) was then calculated using the following formula: PDT = hours of exponential phase/[(logN2–logN1)/log 2], where N1 is the number of cells at the beginning of the exponential growth phase and N2 is the number of cells at the end of the exponential growth phase.

### Evaluation of the osteogenic differentiation potential

To compare the osteogenic differentiation potentials of the cells from the repeated cultures, the expression levels of osteogenic genes and proteins were analyzed by RT-PCR and western blotting (WB), respectively. T1-P3, T3-P3, and T5-P3 cells were cultured with osteogenic medium and harvested after 14 days of cultivation. The RT-PCR procedure was similar to that described above except that 18S RNA was used as the reference gene. The osteogenesis-related genes of interest were runt-related transcription factor 2 (RUNX2), ALP, and osteocalcin. The primers used are listed in Table 1-part 2. For WB, T1-P3, T3-P3, and T5-P3 cells were collected and lysed with SDS lysis buffer (100****mM Tris at pH****8.0, 10% glycerol, and 1% SDS). The protein concentrations were determined using a NanoVue spectrophotometer (GE, USA). Equal amounts of protein were separated by 8%–15% SDS-PAGE (8% for ALP; 12% for RUNX2 and β-actin; and 15% for OC) and then transferred to polyvinylidene fluoride (PVDF) membranes. After blocking with 5% milk, the membranes were incubated overnight at 4°C with the following primary antibodies: anti-RUNX2, anti-ALP, or anti-OC (1∶1000 dilution; Abcam, USA), followed by incubation with horseradish peroxidase-conjugated secondary antibody (1∶2000 dilution; Southern Biotech, Birmingham, AL, USA) at room temperature for 1 hour. The signals were detected by enhanced chemiluminescence (KPL, Gaithersburg, MD, USA). β-actin was used as the loading control.

### Construction of the tissue-engineered bone

Human demineralized bone mass (DBM) was purchased from Beijing Datsing Bio-Tech Co.,Ltd. (BIO-GENE) and manufactured by the modified methods according to the methods described in Xing et al., [3]. The samples were sectioned into cubes (2****mm×2****mm×4****mm), residual lipid tissue was removed, and demineralized. The DBM scaffolds were processed by vacuum freeze-drying, vacuum packaging and irradiation sterilization. They were typically stored at −70°C for at least 3 months before use. TEB was fabricated according to the reference [17]. The T1-P3, T5-P3 hUCMSCs were redissolved in common medium at a cell density of 10^6^ per milliliter. 0.01****mL cell suspensionwas seeded to each side of DBM of two-2****mm×4****mm sides. After 2 hours incubation, the other side of DBM was prepared in the same way. After another 2 hours, the prepared TEB was immersed in osteogenic medium for the further culture for 7 days prior to the surgical implantation.

### Surgical procedure and osteogenic evaluation

TEB containing hUCMSCs and DBM was washed with DMEM medium to remove bovine serum prior to the implantation. 5 nude mice (BALB/Cnu/nu) were anesthetized with sodium pentobarbital (45****mg/kg) (Sigma, St. Louis, MO, USA) by intraperitoneal injection according to approval of the Animal Care and Use Committee of The Third Military Medical University (Chongqing, China). The bilateral iliums were exposed from posterolateral approach. The periosteum was erased by knife, and TEB with T1-P3 and T5-P3 were implanted into the right and left site of defective periosteum, respectively. TEBs were fixed by monofilament polypropylene suture (6-0, Urgalloy, Japan).

On 56d postoperation, nude mice were sacrificed for imaging using X-ray (Mammography system MAMMOMAT 3000 Nova, Siemens, Germany) and micro-CT (Viva CT40, Scanco Medical AG, Bassersdorf, Switzerland). The voltage and beam current of the X-ray source were 50 Kv and 160 mA, respectively, representing the parameters at which mineralized tissue can be visualized best. The volume of new bone were derived from the CT images by delineating the areas of contrast-enhanced regions of interest (ROI) in the X, Y, and Z planes. Images were obtained by adjusting the micro-CT data window display to enhance the edge delineation of the new bonemass. The 3-D images of TEB were reconstructed using a middle-frequency kernel in 1****mm thick axial scanning density. The quantitative measurements of the new bone were used bone volume per tissue volume (BV/TV) and bone mean density (BMD). Then, the samples *in* **** *vivo* were sectioned into small bone blocks for histology according to the reference [17] described below. Samples were fixed in 10% neutral buffered formalin for 48 hours, subjected to decalcification, dehydrated in graded alcohols, and embedded in paraffin. Then samples were sectioned to 5****µm thickness, and stained with Masson trichrome and HE staining. Photomicrographs of sections were taken with an Olympus BX-60 light microscope and an Olympus 3CCD color video camera.

### Statistical analysis

All of the results are presented as the mean ± SD, based on three technical replicates. For the statistical analysis, the RT-PCR and WB values were transformed to natural log values to obtain normally distributed data. The data for the three batches of cells were compared using a one-way ANOVA (SPSS version 13.0). P<0.05 was considered statistically significant.

## Results

### Distribution of MSCs in the UC and rate of successful cell cultivation

The UC microstructure was revealed by HE staining (Fig. 1E–I). As proposed by the mesenchymal and tissue stem cell committee of the International Society for Cellular Therapy (ISCT), cells must be positive for CD73, CD90 and CD105 to be defined as MSCs [17]. Accordingly, in the present study, only the cells carrying these markers were categorized as MSCs. IF staining of freshly excised UCs showed that the CD73^+^, CD90^+^ and CD105^+^ MSCs were mainly located in the Wharton’s jelly (Fig. 1J–L) and that the CD31^+^ endothelial progenitor cells were in the vascular intima. After adherent culture for 7 days, the MSCs had drifted to the margins of the tissue blocks, whereas they were distributed evenly after suspension culture (Figs 1M and 1N). All of the tissue block in the RCF were firmly attached to the bottom, whereas an average of 13 tissue blocks were floating in the CCD (Fig. 1A–D, n = 3). Before the first passage on day 12, the number of viable cells was significantly greater in the RCF (24.7×10^4^±3.6×10^4^, n = 3) than in the CCD (5.1×10^4^±1.0×10^4^, n = 3; p<0.01). Consistently, cultivation using the RCF also remarkably increased the success rate (86±2.7% versus 44±4%, n = 3; p<0.01). Moreover, for cells cultured using the RCF, after the first passage, the time until the next passage declined to 6 days.

### Comparison of phenotypic profiles

**Figure 2:**
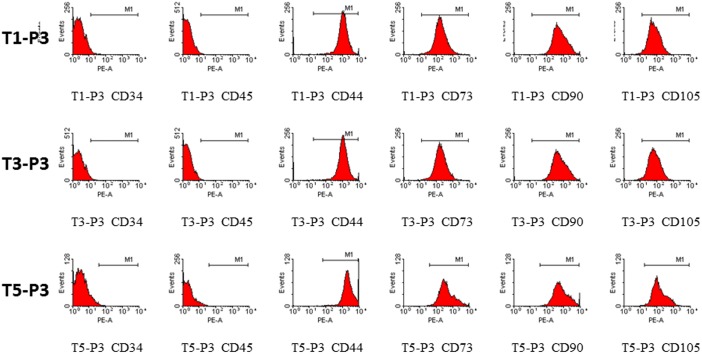
Immunophenotypic profiles of the T1-P3, T3-P3, and T5-P3 cells.

To characterize the cells in the repeated cultures, their immunophenotypes were determined (Fig. 2). Based on flow cytometric analysis, the T1-P3, T3-P3, and T5-P3 cells were negative for CD34 and CD45, indicating that they were nonhematopoietic cell populations. Consistent with the abovementioned standard, all of the T1-P3, T3-P3, and T5-P3 cells were CD73^+^, CD90^+^ and CD105^+^ biomarkers typical of hMSC. In addition, all of these cells were positive for the cell adhesion molecule CD44.

### Comparison of multilineage differentiation capacities

**Figure 3:**
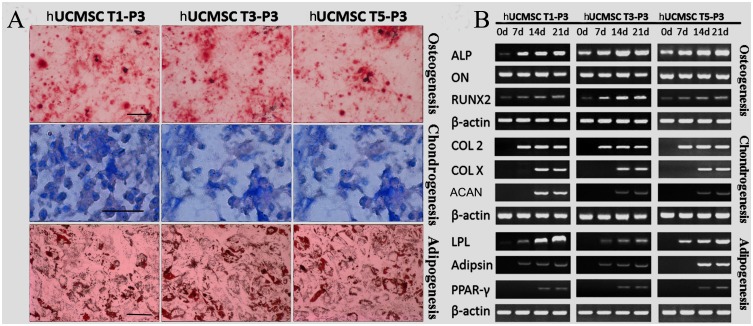
Multilineage differentiation capacities of the T1-P3, T3-P3 and T5-P3 hUCMSCs were confirmed by histochemical staining (A) and RT-PCR (B). (A) The formations of mineralization nodes, lipid-rich vesicles, and a cartilaginous extracellular matrix (sulfated proteoglycans) were revealed by Alizarin red, Oil Red O, and Alcian blue staining, respectively. (B) Additionally, cells were harvested for RT-PCR. LPL, lipoprotein lipase; hUCMSCs, human umbilical cord-derived mesenchymal stromal cells. Scale bars: 25 µm in A.

The multilineage differentiation capabilities of the cells from repeated cultures were tested to evaluate the homogeneity of their lineage. The formation of mineralization nodes, lipid-rich vesicles, and a cartilaginous extracellular matrix (sulfated proteoglycans) were confirmed by Alizarin red, Oil Red O, and Alcian blue staining, respectively (Fig. 3A). Moreover, the osteogenic potentials of the three batches of cells were demonstrated to be uniform, as shown by their expression of osteonectin mRNA and the gradual increase in their ALP and RUNX2 mRNA expression (Fig. 3B). Furthermore, the expression of chondrogenic (COL II, COL X and Agg) and adipogenic marker are positive yet heterogeneously expressed between T1, T3 and T5. (Fig. 3B).

### Comparison of the growth kinetics

**Figure 4:**
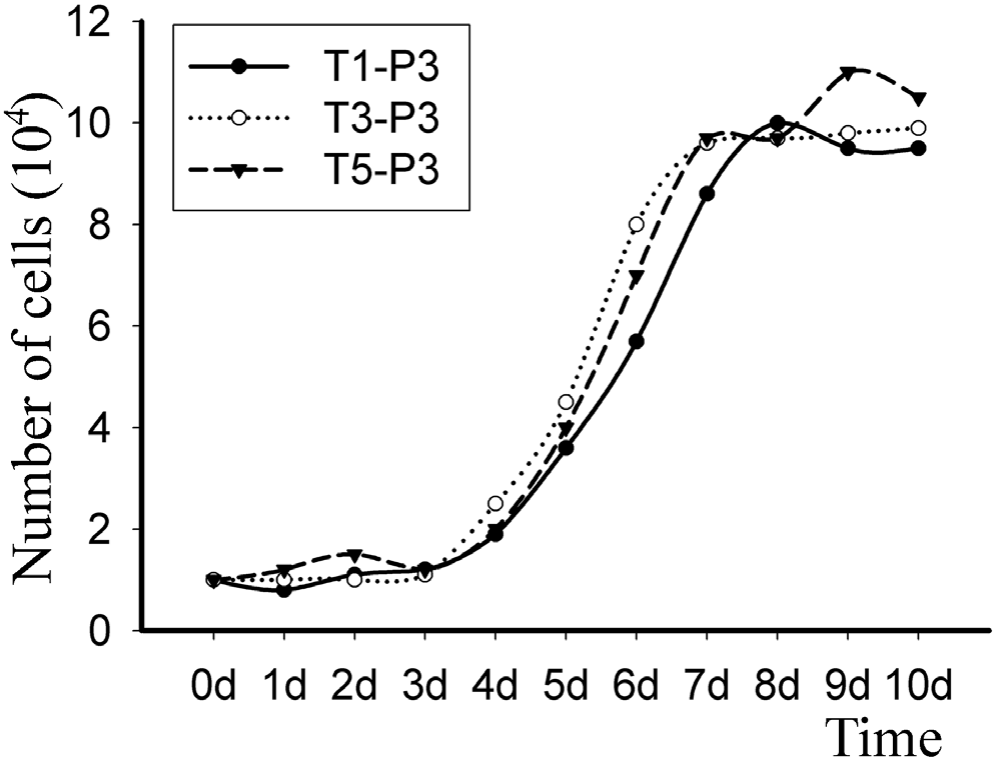
Growth curves of the T1-P3, T3-P3 and T5-P3 hUCMSCs. The growth curves demonstrate the number of days that the cells were in the lag, log and stationary phases of growth.

As shown in Fig. 4, the growth patterns of the T1-P3, T3-P3 and T5-P3 cells exhibited a similar tendency. A short temporary adaptation or lag phase lasted for 3 days, and subsequently, an extensive log phase began and lasted until day 7, when confluence was attained. The plateau phase existed from day 7 onward. The population doubling time (T0) was found to be approximately 32****h for the three batches of cells.

### Further comparison of osteogenesis-related proteins

**Figure 5:**
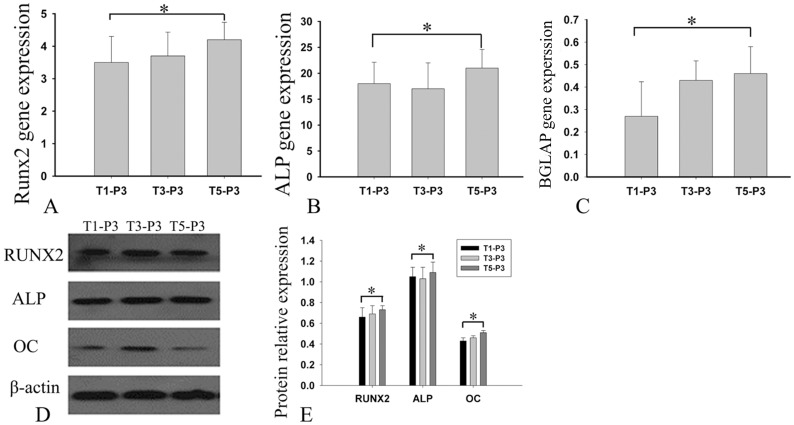
Expression of osteogenic differentiation markers. After a 14-day incubation in osteogenic medium, the expression of osteogenic differentiation markers by T1-P3, T3-P3 and T5-P3 cells was assayed using real-time RT-PCR (A–C). The y-axes (A–C) represent the expression rate of the target genes relative to that of 18S. At the same time, cells were lysed for western blotting. The expression of the osteogenic differentiation markers was detected in the three batches of cells (E). Bars represent the mean ± SD, n = 3. *p>0.05.

Considering the currently urgent need for large amounts of MSCs for bone tissue engineering, the uniformity of the osteogenic capacity was evaluated. Quantification using real-time RT-PCR showed that there was no significant difference in gene expression among the three batches of cells (P>0.5), whether for ALP, RUNX2 or OC (Figs 5A–C). Consistently, WB showed that the levels of ALP, RUNX2, and OC proteins also were not noticeably different (Figs 5D and E).

### Osteogenesis of TEB

**Figure 6:**
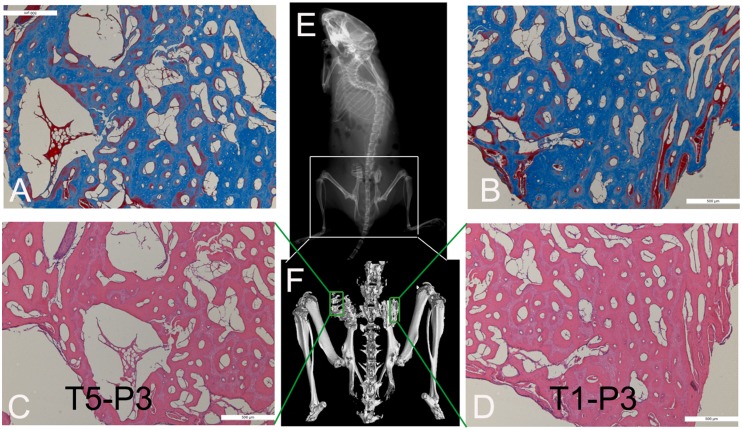
Osteogenic evaluation *in* *vivo* of TEB containing T5-P3 hUCMSCs (A and C) and T1-P3 hUCMSCs (B and D). X-ray (E), micro-CT (F), and staining of Masson (A and B) and HE (C and D)were applied to demonstrat new bone formation and neoformative blood vessels in hibateral implanted TEB. Scale bars: 500 um in A–D.

The osteogenesis of TEB containing hUCMSCs from T1-P3 and T5-P3 was observed on 56d post-implantation (Fig. 6). Both X-ray and micro-CT showed that osteogenesis of hibateral TEB was similar. The value of BV/TV from right implant (TEB with T1-P3 hUCMSCs) and left implant (TEB with T5-P3 hUCMSCs) was (44.2±5.3) % and (48.5±4.4) %, respectively. The value of BMD from them was (0.41±0.09) g/cm^2^ and (0.44±0.08) g/cm^2^, respectively. There was no significant difference between them (p<0.05). The staining of HE and Masson demonstrated new bone formation in hibateral implanted TEB. The osteoblasts and neoformative blood vessels also were observed in implanted TEB.

## Discussion

In bone tissue engineering, it is pivotal but difficult to obtain abundant seed cells of dependable quality. Being derived from the UC, an immunoprivileged organ [18], hUCMSCs have inspiring immunomodulatory effects [19], [20], [21], extensive sources, and a noninvasive preparative process [22], which makes them promising seed cells. However, the current culture methods are not simple and efficient and often fail to make the best use of the UC tissues. Moreover, the culture processes cannot be performed on a large scale and under strict quality control. Thus, we designed an umbilical cord Wharton’s jelly repeated culture system and made efforts to obtain plentiful hUCMSCs of dependable quality during a short culture period. The stained cross-section shows the structure of the UC, which encloses the vessels (two arteries and one vein) in a mass of mucous connective tissue named Wharton’s jelly (WJ) (Fig. 1I) and is lined by the umbilical epithelium [18]. Identified by their cell surface antigens, the majority of MSCs were located in the Wharton’s jelly, and the rest lay in the sub-amnion layer, the perivascular regions, and the vessels, which is why the Wharton’s jelly is called a reservoir of mesenchymal stem cells [23], [24]. In the present study, the amnion layer, vessels and ureters were thoroughly dissected to remove as many of the resident epithelial, endothelial and muscle cells as possible (Figs 1F and G). During this process, the dense connections around the borders of the Wharton’s jelly were also destroyed to help its MSCs migrate into the culture medium. Under such circumstances, the repeated culture of UC tissues was feasible, as confirmed by the highly purified MSCs obtained from the quintic cultures.

To ensure early cell migration, firm adherence of the UC tissues to the bottom of the dish is a prerequisite [25]. We found that most MSCs drifted to the marginal regions of the tissue blocks after adherent culture for 7 days. In contrast, suspension culture led to an even distribution of the MSCs in the block (Figs 1M and 1N). Considering the inherent ability of MSCs to adhere to plastic, the force created by the firm adhesion of the tissue block to the dish might empower cell migration [26], [27], [28]. During conventional adherent culture, it is difficult to keep the tissue block attached to the dish due to their buoyancy and the impact forces of the medium, as well as the inevitable movements that occur during manipulation and observation [9], [29]. Indeed, an average of 13 tissue blocks (26%) was floating in the adherent cultures in a CCD. However, the use of the RCF significantly improved the culture’s efficiency by retaining all of the tissue block on the bottom of the dish. The slots succeeded in conquering the buoyancy caused by moving the dish and changing the medium.

Wharton’s jelly is composed primarily of collagen and mesenchymal cells. The tissue blocks lacking the UC intima and adventitia, vessels and ureter can be repeatedly cultured [30]. When using CCDs for repeated culture, the tissue block must be moved to another dish one by one. While using the RCF, movement of the bracket body was easier than other methods. And RCF could provide a simplified procedure and a reduced probability of contamination. Because the appropriate distance between the tissue block was approximately 1****cm, the concentric steel rings were set 1****cm apart to make proper use of space in the dish. During repeated culture using the RCF, the T1-P3, T3-P3 and T5-P3 cells exhibited a similar fibroblastic morphology. Moreover, it took only 6 days from the first passage to the second, which might be attributed to the concentration of the MSCs in the marginal regions. Additionally, the flow cytometric data indicated no signs of phenotypic changes or cellular impurity with the increasing culture period. Furthermore, the T1-P3, T3-P3 and T5-P3 cells showed a uniform and satisfactory potential for multilineage differentiation. These data collectively characterized the three batches of cells as MSCs of dependable quality.

In particular, the successful application of hUCMSCs from repeated cultures in bone tissue engineering called for comparison of some specific biological properties. The PDT assay revealed that the proliferative capacities of the T1-P3, T3-P3 and T5-P3 hUCMSCs were similar. The reason might be that the hUCMSCs are earlier-stage cells compared with adult MSCs, and the early embryonic antigen SSEA-4 reportedly identifies adult MSC populations [31]. It is well known that during osteogenic differentiation, some of the osteogenic markers, such as ALP [32], RUNX2 [33], [34], and OC, are expressed sequentially. Consistent with their osteogenic differentiation capacity, the levels of ALP, RUNX2, and OC expression were similar in the hUCMSCs from the repeated cultures, indicating that they had an equally remarkable osteogenic potential. Furthermore, osteogenesis of TEB containing repeated cultured hUCMSCs *in* **** *vivo* suggested that there was no significant difference between repeated hUCMSCs. Compared with DBM alone, TEB fabricated with DBM and hUCMSCs of the T1-P3 displayed higher osteogenic capability (see Figure S1), which suggested that hUCMSCs of the T1-P3 palyed an important role in newly formed bone. Some researches demonstrated that transplanted MSCs directedly were involved in newly formed bone [35], [36].

In summary, the application of the RCF in repeated UC cultures simplified and specified the culture process, and the culture efficiency was significantly improved. The goal of acquiring abundant hUCMSCs with dependable quality was achieved with the use of RCF. The present study solved the issue of the sources of seeding cells and helped lay the foundation for the industrialization of bone tissue engineering. Despite the improved prospects, the *in vivo* safety of hUCMSCs obtained from repeated cultures requires further investigation.

## Supporting Information

Figure S1 **Osteogenic evaluation in vivo of TEB containing T1-P3 hUCMSCs (A and B) and DBM alone (C and D).** Micro-CT (E), and staining of Masson (A and C) and HE (B and D) were applied to demonstrate new bone formation and neoformative blood vessels in hibateral implanted TEB. Newly formed bone tissues in TEB group were more and better than that in DBM group on 56d postoperation. Scale bars: 500 um in A–D.
(TIF)

## References

[1] Han D, Li J (2013) Repair of bone defect by using vascular bundle implantation combined with Runx II gene-transfected adipose-derived stem cells and a biodegradable matrix. Cell Tissue Res 352: 561–571. 23604755 10.1007/s00441-013-1595-9

[2] Lee JH, Kim JH, Oh SH, Kim SJ, Hah YS, et al (2011) Tissue-engineered bone formation using periosteal-derived cells and polydioxanone/pluronic F127 scaffold with pre-seeded adipose tissue-derived CD146 positive endothelial-like cells. Biomaterials 32: 5033–5045. 21543114 10.1016/j.biomaterials.2011.03.081

[3] Hou T, Li Q, Luo F, Xu J, Xie Z, et al (2010) Controlled dynamization to enhance reconstruction capacity of tissue-engineered bone in healing critically sized bone defects: an *in vivo* study in goats. Tissue Eng Part A 16: 201–212. 19678758 10.1089/ten.TEA.2009.0291

[4] Kodama A, Kamei N, Kamei G, Kongcharoensombat W, Ohkawa S, et al (2012) *In vivo* bioluminescence imaging of transplanted bone marrow mesenchymal stromal cells using a magnetic delivery system in a rat fracture model. J Bone Joint Surg Br 94: 998–1006. 22733960 10.1302/0301-620X.94B7.28521

[5] Lu Z, Wang G, Dunstan CR, Chen Y, Yenn-Ru Lu W, et al (2013) Activation and promotion of adipose stem cells by tumour necrosis factor-alpha preconditioning for bone regeneration. J Cell Physiol 228: 1737–1744. 23359411 10.1002/jcp.24330

[6] Wang L, Ott L, Seshareddy K, Weiss ML, Detamore MS (2011) Musculoskeletal tissue engineering with human umbilical cord mesenchymal stromal cells. Regen Med 6: 95–109. 21175290 10.2217/rme.10.98 PMC3057462

[7] Longo UG, Loppini M, Berton A, La Verde L, Khan WS, et al (2012) Stem cells from umbilical cord and placenta for musculoskeletal tissue engineering. Curr Stem Cell Res Ther 7: 272–281. 22563663 10.2174/157488812800793054

[8] Hou T, Xu J, Wu X, Xie Z, Luo F, et al (2009) Umbilical cord Wharton’s Jelly: a new potential cell source of mesenchymal stromal cells for bone tissue engineering. Tissue Eng Part A 15: 2325–2334. 19231937 10.1089/ten.tea.2008.0402

[9] Ishige I, Nagamura-Inoue T, Honda MJ, Harnprasopwat R, Kido M, et al (2009) Comparison of mesenchymal stem cells derived from arterial, venous, and Wharton’s jelly explants of human umbilical cord. Int J Hematol 90: 261–269. 19657615 10.1007/s12185-009-0377-3

[10] Leeb C, Jurga M, McGuckin C, Moriggl R, Kenner L (2010) Promising new sources for pluripotent stem cells. Stem Cell Rev 6: 15–26. 20091142 10.1007/s12015-009-9102-0

[11] Liu S, Yuan M, Hou K, Zhang L, Zheng X, et al (2012) Immune characterization of mesenchymal stem cells in human umbilical cord Wharton’s jelly and derived cartilage cells. Cell Immunol 278: 35–44. 23121974 10.1016/j.cellimm.2012.06.010

[12] Wegmeyer H, Bröske AM, Leddin M, Kuentzer K, Nisslbeck AK, et al (2013) Mesenchymal stromal cell characteristics vary depending on their origin. Stem Cells Dev 22: 2606–2618. 23676112 10.1089/scd.2013.0016 PMC3780294

[13] Lönne M, Lavrentieva A, Walter JG, Kasper C (2013) Analysis of oxygen-dependent cytokine expression in human mesenchymal stem cells derived from umbilical cord. Cell Tissue Res 353: 117–122. 23579552 10.1007/s00441-013-1597-7

[14] Salehinejad P, Alitheen NB, Ali AM, Omar AR, Mohit M, et al (2012) Comparison of different methods for the isolation of mesenchymal stem cells from human umbilical cord Wharton’s jelly. In Vitro Cell Dev Biol Anim 48: 75–83. 22274909 10.1007/s11626-011-9480-x

[15] Yoon JH, Roh EY, Shin S, Jung NH, Song EY, et al (2013) Comparison of Explant-Derived and Enzymatic Digestion-Derived MSCs and the Growth Factors from Wharton’s Jelly. Biomed Res Int 2013: 428726. 23653895 10.1155/2013/428726 PMC3638666

[16] Lu LL, Liu YJ, Yang SG, Zhao QJ, Wang X, et al (2006) Isolation and characterization of human umbilical cord mesenchymal stem cells with hematopoiesis-supportive function and other potentials. Haematologica 91: 1017–1026. 16870554

[17] Xing J, Hou T, Luobu B, Luo F, Chen Q, et al (2013) Anti-Infection Tissue Engineering Construct Treating Osteomyelitis in Rabbit Tibia. Tissue Eng Part A 19: 255–263. 22861191 10.1089/ten.TEA.2012.0262

[18] Corrao S, La Rocca G, Lo Iacono M, Corsello T, Farina F, et al (2013) Umbilical cord revisited: From Wharton’s jelly myofibroblasts to mesenchymal stem cells. Histol Histopathol 10: 1235–1244. 10.14670/HH-28.1235 23595555

[19] Najar M, Raicevic G, Boufker HI, Fayyad-Kazan H, De Bruyn C, et al (2010) Adipose-tissue-derived and Wharton’s jelly-derived mesenchymal stromal cells suppress lymphocyte responses by secreting leukemia inhibitory factor. Tissue Eng Part A 16: 3537–3546. 20597819 10.1089/ten.TEA.2010.0159

[20] Balasubramanian S, Venugopal P, Sundarraj S, Zakaria Z, Majumdar AS, et al (2012) Comparison of chemokine and receptor gene expression between Wharton’s jelly and bone marrow-derived mesenchymal stromal cells. Cytotherapy 14: 26–33. 22091833 10.3109/14653249.2011.605119

[21] Anzalone R, Lo Iacono M, Loria T, Di Stefano A, Giannuzzi P, et al (2011) Wharton’s jelly mesenchymal stem cells as candidates for beta cells regeneration: extending the differentiative and immunomodulatory benefits of adult mesenchymal stem cells for the treatment of type 1 diabetes. Stem Cell Rev 7: 342–363. 20972649 10.1007/s12015-010-9196-4

[22] In’t Anker PS, Scherjon SA, Kleijburg-van der Keur C, de Groot-Swings GM, Claas FH, et al (2004) Isolation of mesenchymal stem cells of fetal or maternal origin from human placenta. Stem Cells 22: 1338–1345. 15579651 10.1634/stemcells.2004-0058

[23] Romanov YA, Svintsitskaya VA, Smirnov VN (2003) Searching for alternative sources of postnatal human mesenchymal stem cells: candidate MSC-like cells from umbilical cord. Stem Cells 21: 105–110. 12529557 10.1634/stemcells.21-1-105

[24] Sarugaser R, Lickorish D, Baksh D, Hosseini MM, Davies JE (2005) Human umbilical cord perivascular (HUCPV) cells: a source of mesenchymal progenitors. Stem Cells 23: 220–229. 15671145 10.1634/stemcells.2004-0166

[25] Atala A, Lanza PR (2002) Methods of tissue engineering. New York, Academic Press. 18–19 p.

[26] Lushaj EB, Anstadt E, Haworth R, Roenneburg D, Kim J, et al (2011) Mesenchymal stromal cells are present in the heart and promote growth of adult stem cells in vitro. Cytotherapy 13: 400–406. 21090918 10.3109/14653249.2010.529890

[27] De Bruyn C, Najar M, Raicevic G, Meuleman N, Pieters K, et al (2011) A rapid, simple, and reproducible method for the isolation of mesenchymal stromal cells from Wharton’s jelly without enzymatic treatment. Stem Cells Dev 20: 547–557. 20923277 10.1089/scd.2010.0260

[28] Baer PC, Griesche N, Luttmann W, Schubert R, Luttmann A, et al (2010) Human adipose-derived mesenchymal stem cells in vitro: evaluation of an optimal expansion medium preserving stemness. Cytotherapy 12: 96–106. 19929458 10.3109/14653240903377045

[29] Azari O, Babaei H, Derakhshanfar A, Nematollahi-Mahani SN, Poursahebi R, et al (2011) Effects of transplanted mesenchymal stem cells isolated from Wharton’s jelly of caprine umbilical cord on cutaneous wound healing; histopathological evaluation. Vet Res Commun 35: 211–222. 21340694 10.1007/s11259-011-9464-z

[30] Saward L, Zahradka P (1997) Coronary artery smooth muscle in culture: migration of heterogeneous cell populations from vessel wall. Mol Cell Biochem 176: 53–59. 9406145

[31] Gang EJ, Bosnakovski D, Figueiredo CA, Visser JW, Perlingeiro RC (2007) SSEA-4 identifies mesenchymal stem cells from bone marrow. Blood 109: 1743–1751. 17062733 10.1182/blood-2005-11-010504

[32] Wang DA, Williams CG, Yang F, Cher N, Lee H, et al (2005) Bioresponsive phosphoester hydrogels for bone tissue engineering. Tissue Eng 11: 201–213. 15738675 10.1089/ten.2005.11.201

[33] Ducy P, Zhang R, Geoffroy V, Ridall AL, Karsenty G (1997) Osf2/Cbfa1: a transcriptional activator of osteoblast differentiation. Cell 89: 747–754. 9182762 10.1016/s0092-8674(00)80257-3

[34] Yang S, Wei D, Wang D, Phimphilai M, Krebsbach PH, et al (2003) In vitro and *in* *vivo* synergistic interactions between the Runx2/Cbfa1 transcription factor and bone morphogenetic protein-2 in stimulating osteoblast differentiation. J Bone Miner Res 18: 705–715. 12674331 10.1359/jbmr.2003.18.4.705 PMC3565159

[35] Mankani MH, Kuznetsov SA, Wolfe RM, Marshall GW, Robey PG (2006) *In* *vivo* bone formation by human bone marrow stromal cells: reconstruction of the mouse calvarium and mandible. Stem Cells 24: 2140–2149. 16763200 10.1634/stemcells.2005-0567

[36] Wang F, Yu M, Yan X, Wen Y, Zeng Q, et al (2011) Gingiva-derived mesenchymal stem cell-mediated therapeutic approach for bone tissue regeneration. Stem Cells Dev 20: 2093–2102. 21361847 10.1089/scd.2010.0523

