# Supplementary figures and images for: Umbilical Cord Wharton’s Jelly Repeated Culture System: A New Device and Method for Obtaining Abundant Mesenchymal Stem Cells for Bone Tissue Engineering
Source: PLoS One. 2014 Oct 20;9(10):e110764. doi: 10.1371/journal.pone.0110764 (PMC4203828; doi:10.1371/journal.pone.0110764)

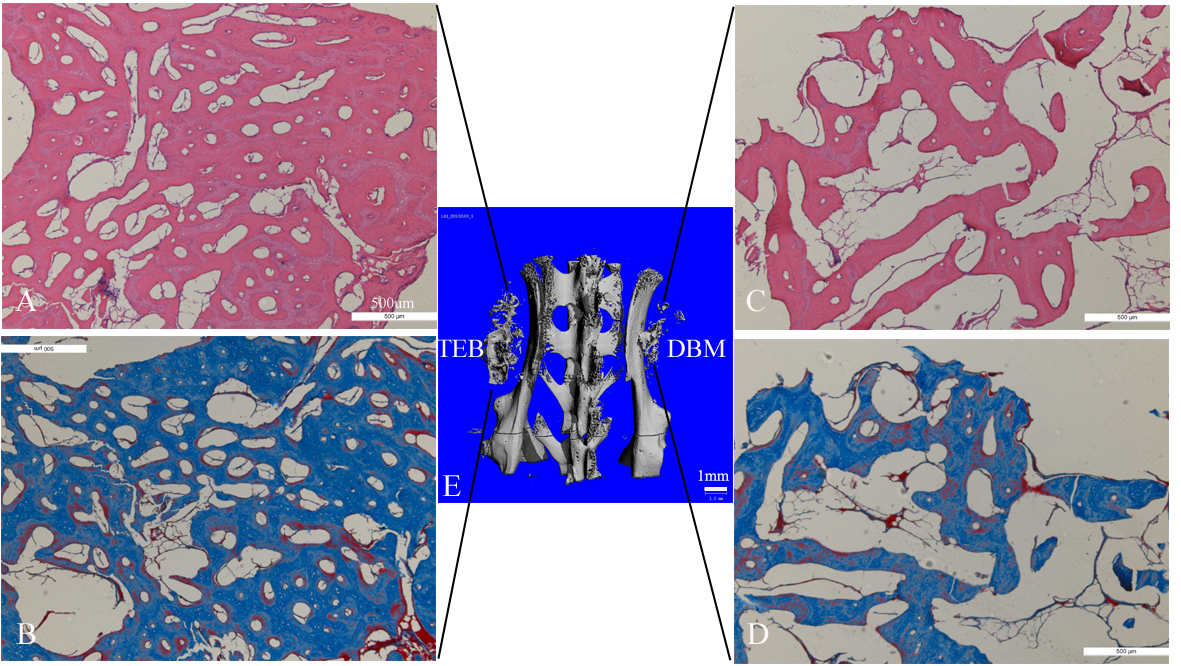

Supplement: Figure S1 — Osteogenic evaluation in vivo of TEB containing T1-P3 hUCMSCs (A and B) and DBM alone (C and D). Micro-CT (E), and staining of Masson (A and C) and HE (B and D) were applied to demonstrate new bone formation and neoformative blood vessels in hibateral implanted TEB. Newly formed bone tissues in TEB group were more and better than that in DBM group on 56d postoperation. Scale bars: 500 um in A–D. (TIF) [file pone.0110764.s001.tif]
